# Right Ventricular Remodeling and Function in Hypoplastic Left Heart Syndrome

**DOI:** 10.1016/j.jacadv.2024.101411

**Published:** 2024-11-20

**Authors:** Thomas Salaets, Marc Gewillig, Alexander Van De Bruaene, Luc L. Mertens

**Affiliations:** a Division of Pediatric Cardiology, University Hospitals Leuven, Leuven, Belgium; b The Labatt Family Heart Centre, Division of Cardiology, The Hospital for Sick Children, Department of Paediatrics, University of Toronto, Toronto, Canada; c Division of Adult Congenital Heart Disease, University Hospitals Leuven, Leuven, Belgium

**Keywords:** congenital cardiology, Fontan, hypoplastic left heart syndrome, single ventricle disease, ventricular remodeling

## Abstract

The right ventricle (RV) in hypoplastic left heart syndrome (HLHS) becomes the systemic ventricle pumping against systemic afterload. It also has to adapt to an initially increased volume load followed by a decrease in volume load after Fontan completion. Anatomical HLHS subtype, therapeutic strategy, tricuspid valve regurgitation, recoarctation, and genetics influence RV size and function. The resulting remodeling process can be maladaptive and lead to ventricular systolic and diastolic dysfunction. While systolic dysfunction is a strong predictor for mortality before Fontan, there is increasing evidence for the impact of progressive diastolic dysfunction after Fontan. This comprehensive review summarizes the (recent) empirical observations that increased understanding of RV remodeling and function in HLHS. It aims at clinicians and researchers wishing to increase their understanding of the physiology of this disease. It highlights the potential for future scientific work on the assessment and preservation of myocardial health throughout the palliation.

## Highlights

• Increased survival of patients with HLHS has led to increased interest in ventricular (mal)adaptive remodeling of the single RV.
• The RV in HLHS remodels in a context of high afterload and initial volume loading followed by volume deprivation.
• Modulating factors include anatomical HLHS subtype, therapeutic strategy, tricuspid valve regurgitation, recoarctation, and genetics.
• Future research on the assessment and preservation of myocardial health throughout the palliation might lead to better clinical outcomes.

Hypoplastic left heart syndrome (HLHS) is a rare form of congenital heart disease that is almost always lethal in the neonatal period if no treatment is offered. About 40 years ago, William Norwood described the first successful stage 1 palliation for patients with HLHS.^1^ Since then, survival of these patients has improved tremendously, and the focus is now on longevity and quality of life. With this shift, remodeling of the right ventricle (RV) in (palliated) HLHS has become a frequently studied topic.

## The normal RV

The normal RV is characterized by a triangular shape in the sagittal plane and a crescent shape on short axis cross-sections. The inflow and outflow components of the RV are separated by the supraventricular crest. Myocardial fibers are organized in two layers: an epicardial circumferential layer and a dominant subendocardial longitudinal layer, with a base to apex orientation.^2^ This fiber organization results in a specific RV contraction pattern, with significant descent of the tricuspid annulus towards the apex and a bellowing effect caused by inward motion of the RV free wall towards the interventricular septum RV function is directly affected by ventricular-ventricular interactions and in the normal RV, contraction of the left ventricle (LV) contributes to RV stroke volume due to contraction of the septum and the presence of shared epicardial fibers encircling both chambers.^3^

During fetal life, the RV is the dominant ventricle. Through the open duct, it is exposed to similar afterload as the LV, while receiving a higher preload. This results in the RV being larger than the LV, with comparable wall thickness. Transition to the normal postnatal circulation involves closure of the duct and decrease in pulmonary vascular resistance leading to progressive thinning of the RV wall.^3^

## How HLHS affects RV structure and function

### RV remodeling in HLHS starts during fetal life

**Figure 1:**
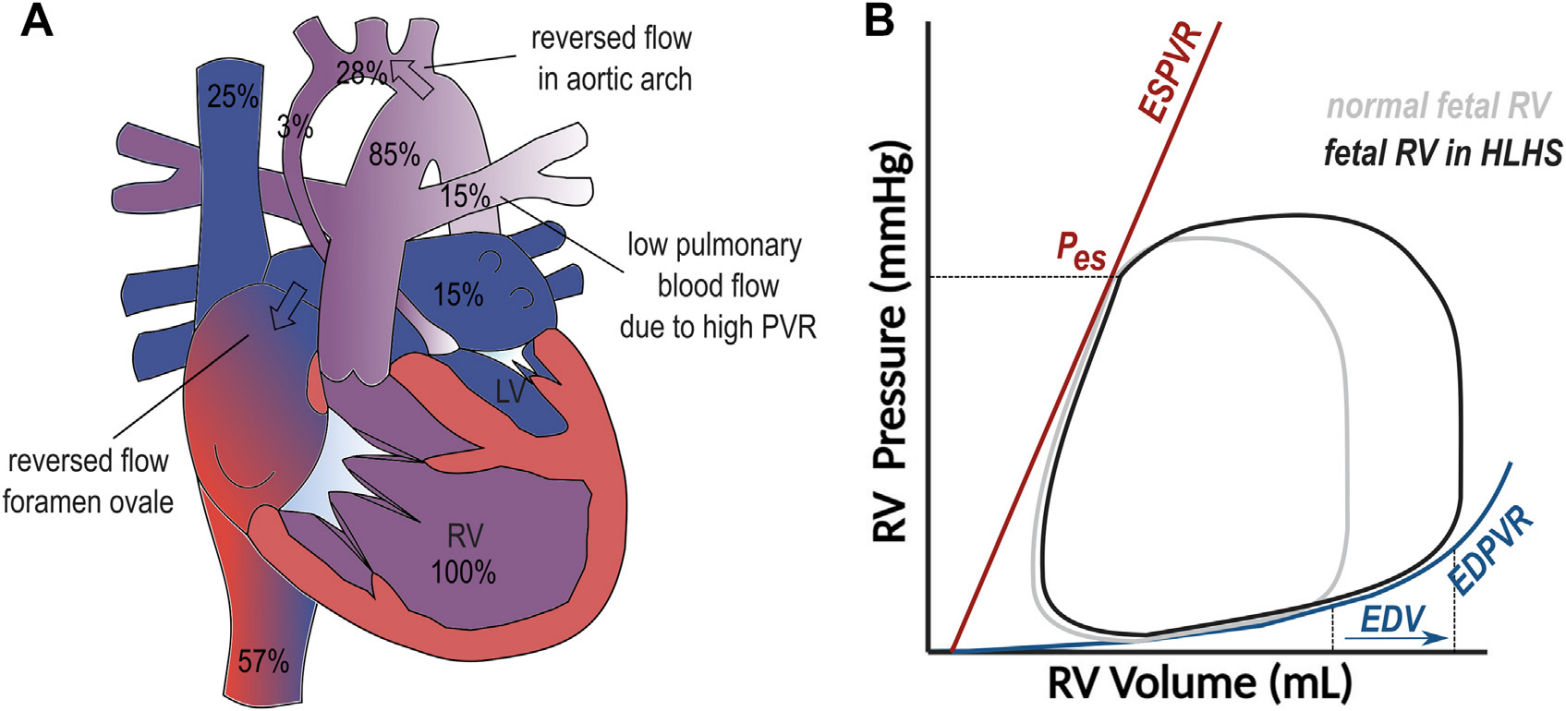
**Fetal HLHS** (A) Anatomical diagram. (B) Hypothetical PV loop. Full figure legend and justification of PV loop can be found in the Supplemental Appendix.

In fetal HLHS, the LV is too small to contribute significantly to the systemic circulation with reversed flow in the aortic arch. In the most extreme cases (mitral and aortic atresia), the RV sustains 100% of the systemic circulation through the arterial duct, including the upper part of the body and the coronary circulation, with blood with an overall lower oxygen saturation.^4^ It receives a higher preload as the normal right to left shunt across the foramen ovale is reduced or reversed (Figure 1).

Remodeling of the RV already occurs in fetuses with HLHS. While the RV has a comparable length as in normal fetuses, it has a larger transverse diameter, making it larger and more spherical.^5^ RV end-diastolic volume (EDV) measured with 4D fetal ultrasound is about 50% higher in HLHS versus normal hearts, independent of the size of the hypoplastic LV.^5^^,^^6^ Interestingly, RV wall thickness was found to be increased in HLHS, despite the pressure being similar,^6^ likely due to a fetal myocardial hyperplastic response to increased volume loading.

Also, myofiber orientation is altered. Using diffusion tensor imaging disorganized fibers with more horizontal but very dispersed organization were noted in samples from neonates and teenagers with HLHS.^7^^,^^8^ Recent data shows that this abnormal pattern is already present in fetuses in HLHS.^9^ Although it is unclear what causes this phenomenon, it is likely to influence RV mechanics.

Fetal RV output in HLHS is increased; however, combined LV and RV cardiac output is lower in HLHS than normal controls.^10^ Interestingly, the RV contraction pattern is altered in utero, with decreased measures of RV longitudinal function but increased radial or circumferential function.^5^^,^^11^

Additionally, there is data that suggests that diastolic function is already affected before birth. There are increased tricuspid valve inflow velocities, with increased dependence on the atrial kick.^5^^,^^11^ Increased inferior vena cava A-wave reversal suggests higher filling pressures.^5^

### Volume loading of the RV after birth and during interstage I

After birth, the single RV must continue to supply both circulations. This requires the presence of a patent ductus arteriosus with obligatory right-left shunting to maintain systemic perfusion and an unrestrictive atrial communication to decompress the left atrium and allow sufficient mixing of saturated blood. Physiological decline in pulmonary vascular resistance will further increase volume loading and stroke volume of the RV. The natural history of this condition is demise within the first weeks of life. Neonatal management consists of maintaining duct patency with prostaglandins and perform the Norwood palliation within the first days of life.

**Figure 2:**
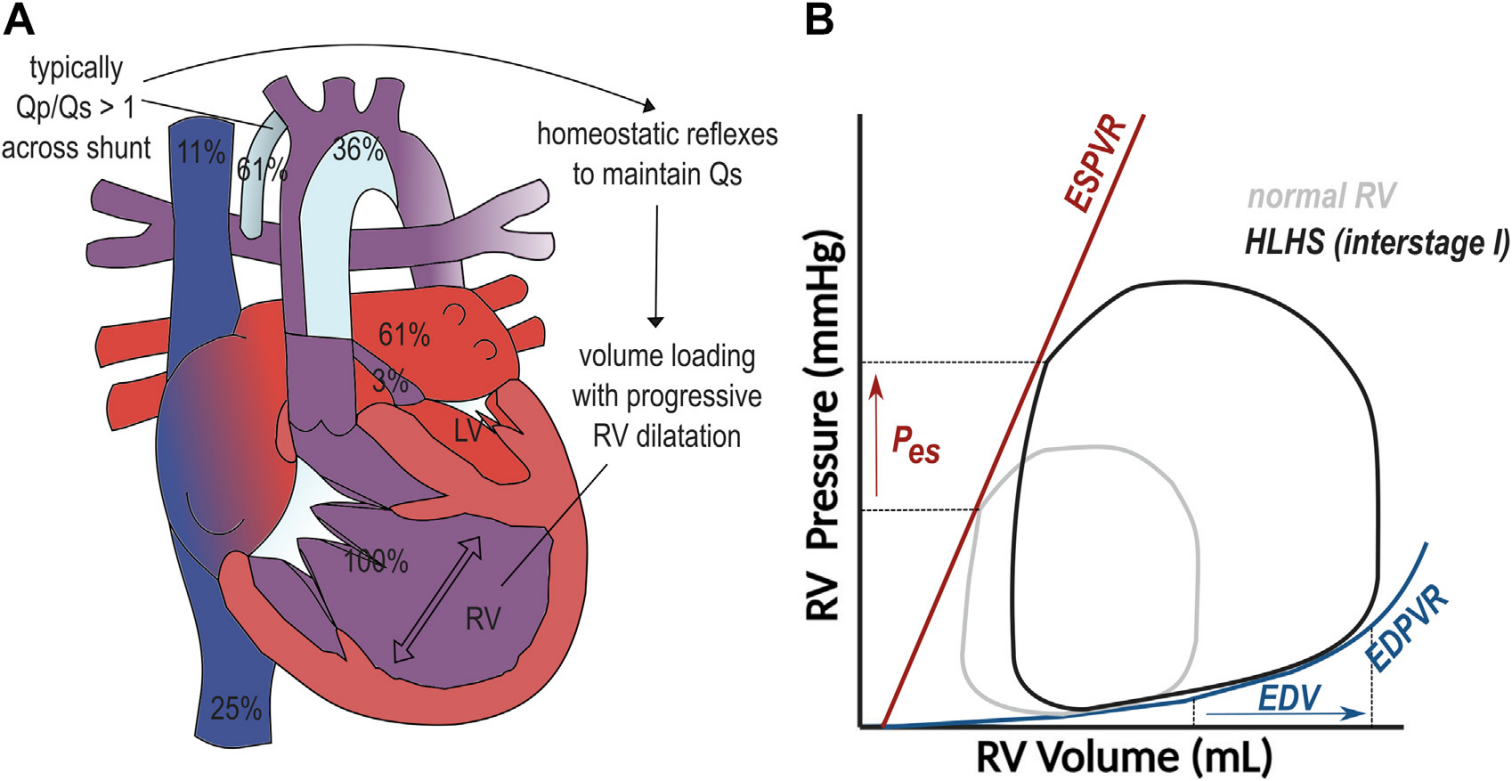
**HLHS After Norwood** (A) Anatomical diagram. (B) Hypothetical PV loop. Full figure legend and justification of PV loop can be found in the Supplemental Appendix.

The Norwood palliation results in the RV remaining the systemic ventricle pumping blood into the reconstructed aorta, while pulmonary blood flow is supplied and controlled either by a modified Blalock-Taussig-Thomas shunt (BTTS) or an RV to pulmonary artery conduit (Sano modification or RV-PA conduit) (Figure 2). This maintains RV pressure at systemic levels. During the months after the Norwood palliation (interstage I), average Qp/Qs-values are 1.3 to 1.7 (±1) to 1, which also results in continued volume loading of the RV.^12^

This leads to progressive dilation of the single systemic RV. Increasing indexed RV volumes have been demonstrated between birth and the end of interstage I.^13^^,^^14^ By the end of interstage I, mean indexed EDV has been estimated at 110-160 ml/m^2^. Sphericity of the RV increases further in this stage.^15^

While in the normal heart, the RV walls get thinner during the first months of life, in HLHS patients, RV hypertrophy further increases during interstage I.^14^ Autopsy data from interstage deaths typically show RV myocyte hypertrophy and fibrosis, most pronounced in those with an RV-PA conduit.^16^ Moreover, cellular and genetic expression data suggest changes in gene expression including genes involved in the TGFβ/BMP pathway^17^ and persistence of fetal gene expression patterns.^18^

RV ejection fraction (EF) decreases during interstage I to an average of 46% as measured by 3D echocardiography, with slightly higher values measured by magnetic resonance imaging (50%-58%).^13^^,^^19^^,^^20^ In the interstage, RV longitudinal function further decreases, but circumferential function increases.^15^^,^^21^^,^^22^ The contraction pattern becomes more similar to the LV,^21^ which is in line with the more circumferential RV myofiber organization.^7^

### Unloading the RV after BCPC and TCPC

**Figure 3:**
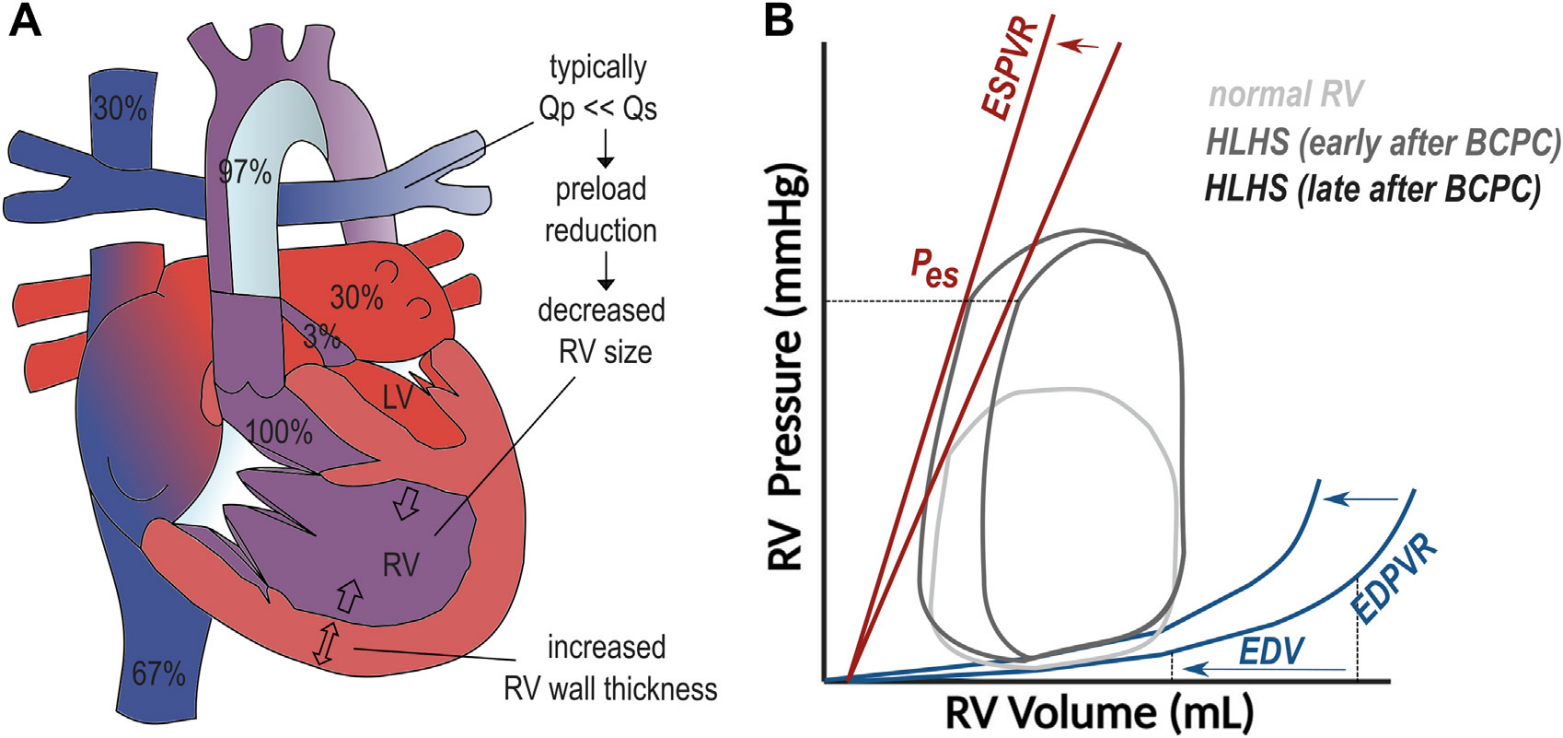
**HLHS After BCPC** (A). Anatomical diagram. (B) Hypothetical PV loop early and late after BCPC. Full figure legend and justification of PV loop can be found in the Supplemental Appendix.

**Figure 4:**
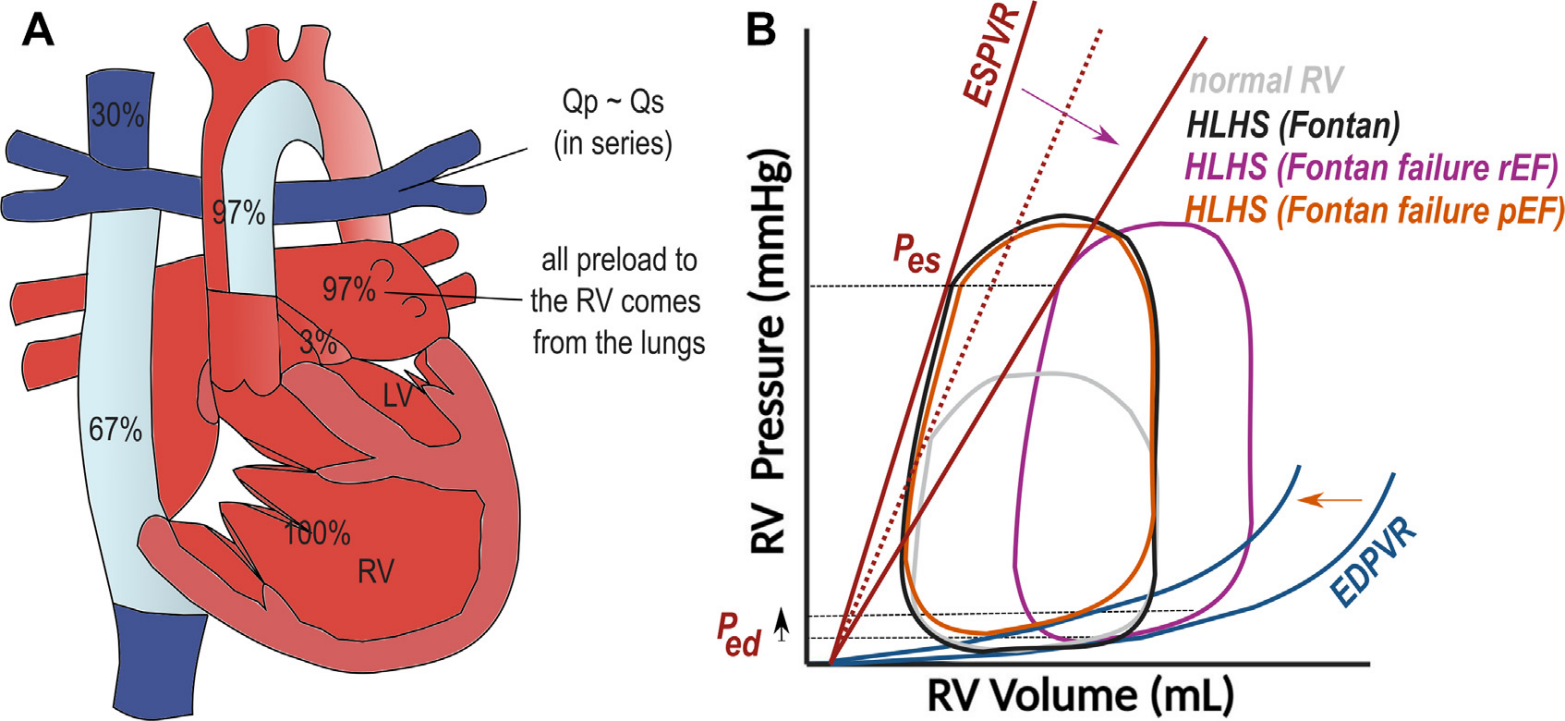
**HLHS Late After TCPC** (A) Anatomical diagram. (B) Hypothetical PV loop, including PV loops after Fontan failure with preserved EF (pEF) and reduced EF (rEF). Full figure legend and justification of PV loop can be found in the Supplemental Appendix.

During staged palliation, the RV is progressively volume unloaded. In the second surgery—typically performed between 4 to 6 months of life—the superior vena cava is directly connected to the pulmonary artery (bidirectional cavopulmonary connection, BCPC or Glenn operation) (Figure 3). In the third stage—performed at around 3 years of age—the inferior vena cava is connected to the pulmonary artery by an extracardiac conduit (total cavopulmonary connection, TCPC or Fontan operation). The redirection of systemic venous blood unloads the RV which is now filled by pulmonary venous blood only. The systemic and pulmonary circulations thus function in series again without subpulmonary pump.^23^ Meanwhile, the systemic RV continues to be pressure loaded (Figure 4).

In historic series after nonstaged Fontan completion, important and acute volume reductions have been observed (−52%).^24^ Nowadays, these changes in ventricular volume are more insidious, with a reduction of 11% in RV EDV, only at the end of interstage II.^25^

Measures of RV function decrease early after BCPC, which could reflect the acute decrease in preload.^13^^,^^26^ At the end of interstage II, however, noninvasive markers of RV systolic function improve again. The relationship between EF and RV volume (“Frank-Starling curve”) shifts leftward in this period, suggesting successful adaptation of contractility to lower levels of preload.^25^ This is in line with an increase in calculated end-systolic elastance, a load independent parameter of contractility, over the same time period.^27^

In early experiences, volume unloading after single step TCPC resulted in an acute increase in relative wall thickness and mass to volume ratio.^24^ The RV muscle needs to rearrange around a smaller cavity and is continuously exposed to high afterload. In historic patient cohorts, this resulted in significant temporary diastolic dysfunction with tachycardia and low cardiac output immediately after the surgery.^28^ Between the pre-BCPC and pre-TCPC assessment, however, end-diastolic ventricular pressures decrease, likely due to the decrease in preload.^29^

### A chronically volume-deprived and pressure-loaded ventricle in the older Fontan patient

After Fontan completion, pulmonary vascular resistance becomes the major controller (or ‘critical bottleneck’) of cardiac output in a Fontan circulation.^23^ In the absence of a driving force for the pulmonary circulation, Fontan patients have difficulty to increase preload and thereby stroke volume of their systemic ventricle during exercise.^30^ Additionally, after aortic arch reconstruction, vascular stiffness is reported to be increased beyond normal levels after Fontan.^31^ For the rest (most) of its lifespan, the RV in HLHS will therefore function under reduced preload and high afterload conditions (Figure 4).

On pressure-volume loops (PV loops), there seems to be a normal coupling of end-systolic ventricular elastance to the increased arterial elastance (afterload) in children with HLHS after Fontan.^27^^,^^32^ The increase in contractility goes together with RV hypertrophy that comes at the expense of decreased RV compliance and reduced diastolic filling.^32^^,^^33^ Especially those patients with recurrent arch obstruction after surgical repair (recoarctation) have decreased RV compliance.^32^

After TCPC, longitudinal strain remains reduced, but also circumferential strain and torsion in the single RV in HLHS remain lower than what is seen in single morphological LV’s.^34^ A more circumferential contraction (LV-like) pattern however was associated to better EF.^35^ In comparison to normal RV’s and LV’s, the single RV in HLHS often displays arguments for dyssynchronous contraction, but the functional importance of this remains uncertain.^36^

After Fontan, there is evidence of progressively increasing RV volumes, with progressive decrease in RV EF and systolic strain parameters.^37^^,^^38^ The increase in RV volume could be an early sign of worsening contractility or could reflect volume loading due to increasing tricuspid valve regurgitation and collateral burden.

Finally, the post-Fontan single RV has multiple reasons to develop diastolic dysfunction and increased filling pressures (Figure 4). First of all, the adaptation of the RV to chronic pressure loading involves a hypertrophic response that makes the muscle less compliant. Additionally, volume unloading might increase mass to volume ratio and increased relative wall thickness. Pathologic remodeling and relative ischemia can cause diffuse fibrosis affecting compliance.^39^ Finally, also decreases in contractility (induced by remodeling, ischemia, or scarring) result in dilating ventricles operating on higher end-diastolic volumes but also end-diastolic pressures on the PV loop.

While RV diastolic dysfunction is likely common in older Fontans, it is difficult to diagnose noninvasively. Conventional echocardiographic markers such as inflow and tissue Doppler velocities are influenced by the altered preload conditions making their interpretation challenging, with poor correlation with invasively measured RV filling pressures.^40^

## Determinants of RV remodeling and function throughout the lifespan

### HLHS subtype and influence of LV morphology

Three HLHS subtypes with intact septum have been described with variable LV size and LV myocardial properties. The presence of a high-pressure hypertrophic and fibrotic LV can affect RV function through ventriculo-ventricular interactions. A larger and more globular LV is associated with decreased strain in the basal- and mid-septal segments.^41^ There are however conflicting data on whether this negatively affects global RV systolic function.^42^ Larger and stiffer LV cavities however influence RV compliance and could negatively affect RV filling.^43^ More (longitudinal) research is needed, as currently no studies have evaluated the difference in outcome beyond childhood.

### Shunt-type: Modified BTTS versus RV-PA conduit

A classical Norwood includes a modified BTTS, from the brachiocephalic trunk to the central PA. The alternative is an RV-PA conduit which is implanted in the anterior wall of the RV through a small ventriculotomy. The single ventricle reconstruction trial randomized between both approaches, and while there was a small early survival benefit after RV-PA conduit, there were no significant survival differences at longer term.^44^ Both options can have a different effect on RV function.

By the pre-Fontan assessment, there was a small but significant decrease in RV EF in the RV-PA conduit group (44%-41%), but not in the BTTS-group.^45^ This is in line, with longitudinal observational data, indicating a higher proportion of (mainly mild) RV dysfunction in the RV-PA conduit group at longer term after Fontan.^46^ Magnetic resonance data also indicate lower global strain values and slightly more spherical ventricles in comparison to patients with BTTS.^20^ At a microscopic level, more fibrosis has been reported in HLHS RV’s after an RV-PA conduit.^16^ Overall, these functional differences might be statistically significant, they are very small and their relevance for longterm outcomes are unclear.

### Cardiopulmonary bypass

An alternative stage I option is the hybrid approach, which consists of stenting of the arterial duct and bilateral pulmonary artery banding. This strategy avoids cardiopulmonary bypass in the neonatal period. Nevertheless, echocardiographic parameters of RV function often decrease significantly after the hybrid palliation.^47^ At pre-Fontan, magnetic resonance data suggested a slightly lower EDV with no differences in RV EF in the hybrid group compared to Norwood patients.^48^ Overall, the data do not support the hypothesis that a hybrid strategy improves long term RV function.

### Tricuspid valve regurgitation

Tricuspid valve regurgitation is common in patients with HLHS. Due to dilation of the RV in interstage I, there is potential for secondary tricuspid valve regurgitation.^49^ Additionally, the tricuspid valve is structurally abnormal in more than 30% of the HLHS patients. The leaflets are dysplastic, more tethered, more prolapsing, and the anterior papillary muscle tends to be displaced laterally.^49^ Regurgitation affects RV remodeling as it maintains dilation. The size of the ventricle and the tricuspid valve annulus does not decrease after unloading at stage II in those with significant regurgitation.^50^ The effect of regurgitation on RV remodeling seems modifiable by valve surgery.^51^ Most centers aggressively address valve dysfunction through the different stages using a combination of surgical strategies; however, the long term outcome of tricuspid valve surgery in HLHS remains uncertain.

### Recoarctation

Recoarctation after Norwood adds additional pressure loading on the single RV and negatively affects RV remodeling and function. There is an association between smaller or stiffer aortic arches and RV systolic function.^52^ Patients with recoarctation have a high likelihood of developing RV dysfunction and progressive tricuspid valve regurgitation.^53^ Early diagnosis and treatment is therefore important. RV function typically recovers after early balloon dilation of the arch,^53^ but increased arterial elastance and end-diastolic elastance (through hypertrophic remodeling) have a negative impact on Fontan hemodynamics at long term.^32^

### Genetic variations

To date, the etiology of HLHS remains uncertain. For a long time, the most common hypothesis was a structural defect (absent foramen ovale or valvar lesions) leading to abnormal flow distribution with secondary hypoplasia of the LV and remodeling of the RV. An alternative explanation is the presence of a pathogenic mutation that does not only affect the left sided heart structures but also directly influence RV remodeling and function. Whole genome sequencing approaches will probably lead to the discovery of more genetic variants that mediate RV remodeling in HLHS.^54^

## Importance of RV function during HLHS palliation

Studies looking at the early stages of the palliation (after birth or during interstage I) have yielded significant associations between death or transplantation and many RV systolic function variables.^55^, ^56^, ^57^, ^58^ The high event rates in this patient group have undoubtedly increased the statistical power of these relatively small studies. After unloading of the ventricle, the altered loading conditions result in different relationships between ventricular function and outcomes. In the period immediately after BCPC, qualitative dysfunction or decrease in RV fractional area change and RV strain have been shown to be associated to death, transplant, and even later Fontan failure.^22^^,^^26^^,^^59^ However in survivors, ventricular dilation, tricuspid valve regurgitation, and systolic function do improve over time after BCPC,^55^ also in the ones that had prior mildly lower RV functional parameters.^59^ Overall, the mortality becomes much lower after the first year of life.

The relationship between RV function and mortality after Fontan completion is not straightforward as Fontan failure is more complex. There is conflicting empirical evidence on whether systolic functional parameters predict late mortality or transplant after Fontan completion.^60^^,^^61^ This is probably because there are several phenotypes of Fontan failure, with only a (small) subset being heart failure with reduced EF. Other causes of Fontan failure include circulatory or lymphatic failure related to high central venous pressures and low cardiac output in the presence of preserved EF.^62^ Increased ventricular filling pressures have been shown to be associated to death or transplantation in Fontan patients.^63^ Progressive diastolic dysfunction through adverse ventricular remodeling might thus be a major determinant of long-term Fontan prognosis. Besides systolic and diastolic function, also geometry has a prognostic value as indexed ventricular volumes below 125 ml/m^2^ˆ^1.3^ predict transplant-free survival post Fontan.^64^ Finally, also ventricular dyssynchrony has been associated to poor outcomes.^65^

The observed changes in the RV are not only important for prognostication but could also be used as a therapeutic target. They are for instance the target of trials with stem cell therapy in HLHS.^66^ The important association of markers of RV dysfunction or remodeling to prognosis indicates that further research on medical and surgical management strategies to improve myocardial health could lead to improved HLHS outcomes.

## Conclusions

The RV in hypoplastic left heart syndrome takes on a role that is completely different from the normal RV. In this review, we summarized the physiology of the various stages of the palliation and how they affect myocardial remodeling and function. In contrast to the normal RV, the RV in HLHS has to pump against systemic afterload. Additionally, the RV has to adapt to an initial high volume load followed by a low preload state. Moreover several longitudinal variables (anatomic HLHS-subtype, tricuspid valve regurgitation, recoarctation, shunt-type, and genetic factors) influence how the RV copes with these abnormal circumstances throughout the lifespan (Central Illustration). A better understanding of how HLHS and its palliation affect (adverse) myocardial remodeling could lead to a better understanding of the determinants of poor prognosis in this disease.

**Central illustration:**
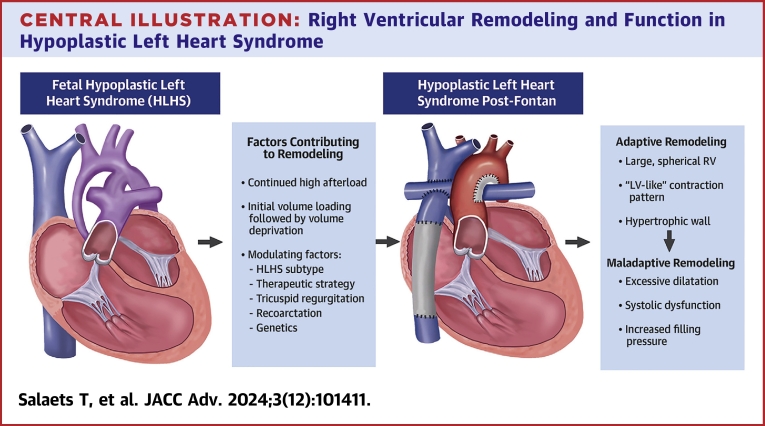
**Right Ventricular Remodeling and Function in Hypoplastic Left Heart Syndrome** From fetal life onwards, the RV in patients with HLHS remodels in response to the specific hemodynamic conditions and patient specific modulators. This results in extraordinary geometrical, mechanical, and biological adaptations that might become maladaptive at various stages of the Fontan palliation.

## Funding support and author disclosure

Dr Salaets was supported for this work by an international ‘long stay’ grant from the 10.13039/501100003130Research Foundation Flanders (FWO V401622N), by the Frans Van de Werf Fund for Clinical Cardiovascular Research (2022 awardee) and by ‘VZW De Kleine Hartjes’. All other authors have reported that they have no relationships relevant to the contents of this paper to disclose.
