# Supplementary material for: Right Ventricular Remodeling and Function in Hypoplastic Left Heart Syndrome
Source: JACC Adv. 2024 Nov 20;3(12):101411. doi: 10.1016/j.jacadv.2024.101411 (PMC11616046; doi:10.1016/j.jacadv.2024.101411)
Supplement: Supplemental material [file mmc1.docx]

**SUPPLEMENTAL APPENDIX**

**Full Figure Legends**

**Supplemental Figure 1. Fetal HLHS.** A. Anatomical diagram. In a typical case with aortic atresia the RV pumps 100% of the combined cardiac output. The flow on the foramen ovale reverses from left to right. The RV ejects all blood in to the pulmonary artery however through a large duct this mostly distributes to the systemic circulation (with reversed flow in the aortic arch for the upper body). The pulmonary blood flow is low due to high pulmonary vascular resistance (PVR). Flow is indicated as percentage of combined cardiac output, while color qualitatively represents blood oxygen saturation (red = high oxygen saturation, blue = low oxygen saturation), based on data from^1^. B. Hypothetical PV loop for the RV in fetal HLHS. End-systolic pressures (P_es_) are unchanged, as the RV continues to pump against systemic vascular resistance through the duct. Preload, and thereby end-diastolic volume (EDV) and stroke volume are increased^2^. For this figure we assume unchanged end-systolic (ESPVR or contractility) and end-diastolic pressure-volume relations (EDPVR or compliance).

**Supplemental Figure 2. HLHS after Norwood.** A. Anatomical diagram. The arch is reconstructed and connected to the pulmonary valve, while pulmonary blood flow is provided through a BTTS (in this drawing) or RV-PA conduit. Given the typically increased Qp/Qs^3^ and the homeostatic reflexes of the body to maintain Qs in absolute numbers, the RV is volume loaded, which will lead to ventricular dilation^4^. Flow is indicated as a percentage of combined cardiac output, while color qualitatively represents blood oxygen saturation (red = high oxygen saturation, blue = low oxygen saturation). For flow percentages we assumed a Qp/Qs of 1.7 to 1, unchanged relationships between upper and lower body in comparison to fetal flow distributions, and unchanged relative coronary blood flow. B. Hypothetical PV loop for the RV in HLHS after Norwood. While the normal RV is exposed to low end-systolic pressures (P_es_) due to the physiological decrease in PVR, the RV in HLHS works against systemic pressures. Additionally there is high preload (due to the high Qp/Qs and the homeostatic reflexes to maintain Qs) which leads to an increase in end-diastolic volume (EDV). For this figure we assume unchanged end-systolic (ESPVR or contractility) and end-diastolic pressure-volume relations (EDPVR or compliance).

**Supplemental Figure 3: HLHS after BCPC.** A. Anatomical diagram. Through the BCPC, the blood flow from the SVC is rerouted to the pulmonary arteries, while the IVC continues to fill the RA. This results in an important decrease in pulmonary blood flow (only 30-50% of total blood flow). This reduces pulmonary venous return and ventricular preload. As a consequence ventricular size decreases and wall thickness increases^5^. Flow is indicated as a percentage of combined cardiac output, while color qualitatively represents blood oxygen saturation (red = high oxygen saturation, blue = low oxygen saturation). For flow percentages we assumed unchanged relationships between upper and lower body and unchanged relative coronary blood flow. B. Hypothetical PV loop of the RV in HLHS after BCPC. Immediately after BCPC the decrease in preload and compliance (shifted end-diastolic pressure volume relationship; EDPVR; due to increased relative wall thickness) result in lower end-diastolic volumes (EDV) and reduced stroke volume. Later after BCPC there are indirect indications that the end-systolic pressure volume relation (ESPVR or contractility curve) increases to compensate and restore stroke volume in a low preload context^5^. Meanwhile the end-systolic pressures remain at systemic levels.

**Supplemental Figure 4: HLHS late after TCPC.** A. Anatomical diagram. After TCPC also the IVC blood flow is rerouted to the pulmonary arteries and all systemic venous blood flow passes through the lungs without subpulmonary pump. The PVR becomes the ‘critical bottleneck’, limiting the preload on the ventricle and cardiac output. Flow is indicated as a percentage of combined cardiac output, while color qualitatively represents blood oxygen saturation (red = high oxygen saturation, blue = low oxygen saturation). For flow percentages we assumed unchanged relationships between upper and lower body and unchanged relative coronary blood flow. B. Hypothetical PV loop of the RV in HLHS after Fontan. The RV functions under high end-systolic pressure (P_es_) and a preload limited by the flow through the lungs. There is empirical data supporting good contractility (ESPVR or end-systolic pressure volume relation), that matches the high afterload in children after Fontan. This adaptation, especially after recoarctation intervention, comes at the cost of decreased compliance (upward shift of end-diastolic pressure volume relation (EDPVR)^6^. Two scenarios of Fontan failure due to adverse ventricular remodeling are illustrated. Both will limit the flow through the Fontan construct by increasing downstream end-diastolic pressures (P_ed_). In Fontan failure with preserved ejection fraction (pEF) decreased compliance (upward shift of EDPVR) results directly in higher P_ed_. In Fontan failure with reduced ejection fraction (rEF) reduced contractility (rightward shift of ESPVR) will also lead to a compensatory increase in end-diastolic volume and pressure (P_ed_) .

**Supplemental References**

1. Sun L, van Amerom JFP, Marini D, et al. MRI characterization of hemodynamic patterns of human fetuses with cyanotic congenital heart disease. *Ultrasound Obstet Gynecol*. 2021;58(6):824-836. doi:10.1002/UOG.23707

2. Zebhi B, Wiputra H, Howley L, et al. Right ventricle in hypoplastic left heart syndrome exhibits altered hemodynamics in the human fetus. *J Biomech*. 2020;112:110035. doi:10.1016/J.JBIOMECH.2020.110035

3. Primeaux J, Salavitabar A, Lu JC, Grifka RG, Figueroa CA. Characterization of Post-Operative Hemodynamics Following the Norwood Procedure Using Population Data and Multi-Scale Modeling. *Front Physiol*. 2021;12. doi:10.3389/FPHYS.2021.603040

4. Kutty S, Graney BA, Khoo NS, et al. Serial assessment of right ventricular volume and function in surgically palliated hypoplastic left heart syndrome using real-time transthoracic three-dimensional echocardiography. *J Am Soc Echocardiogr*. 2012;25(6):682-689. doi:10.1016/J.ECHO.2012.02.008

5. Bellsham-Revell HR, Tibby SM, Bell AJ, et al. Serial magnetic resonance imaging in hypoplastic left heart syndrome gives valuable insight into ventricular and vascular adaptation. *J Am Coll Cardiol*. 2013;61(5):561-570. doi:10.1016/J.JACC.2012.11.016

6. Schlangen J, Fischer G, Petko C, et al. Arterial elastance and its impact on intrinsic right ventricular function in palliated hypoplastic left heart syndrome. *Int J Cardiol*. 2013;168(6):5385-5389. doi:10.1016/J.IJCARD.2013.08.052
